# A Co-Blended and Compounded Photosensitive Resin with Improved Mechanical Properties and Thermal Stability for Nail Polish Application

**DOI:** 10.3390/polym17010040

**Published:** 2024-12-27

**Authors:** Zhihong Chen, Shengsen Wang, Shengyue Feng, Yingzi Huang, Yang Hu, Zhuohong Yang

**Affiliations:** 1 Nanxiong Ketian Chemical Co., Ltd., Shaoguan 512400, China; 13825576610@139.com; 2 Key Laboratory for Biobased Materials and Energy of Ministry of Education, College of Materials and Energy, South China Agricultural University, Guangzhou 510642, China; vincent06266@163.com (S.W.); 13543163820@163.com (S.F.); hyz58011@163.com (Y.H.); huyang0303@scau.edu.cn (Y.H.)

**Keywords:** epoxy soybean oil, photosensitive resins, acid anhydride derivatives, crossing networks

## Abstract

UV-curable bio-based resins are widely used in the UV curing field. However, the current UV-curable bio-based resins for the application of nail polish still have the problems of too high viscosity and insufficiently excellent mechanical properties. In this study, a soybean oil-based acrylate photosensitive resin is synthesized by using epoxidized soybean oil as a raw material and reacting it with acrylic acid. The results show that the viscosity of soybean oil-based acrylate can achieve 8.31 Pa∙s, and the UV-cured film prepared by soybean oil-based acrylate and anhydride derivatives can obtain a tensile strength of 35.36 MPa and an elongation at break of 67.8%. In addition, the soybean oil-based acrylate is further reacted with isophorone diisocyanate to obtain soybean oil-based polyurethane acrylate, which can be thermally stable at 90 °C for 7 d. And then, the UV-cured film constructed by soybean oil-based polyurethane acrylate and anhydride derivatives are prepared, and the elongation at the break of the cured films can be up to 320%. This work provides a solvent-free approach by using biomass raw materials to form polyurethane acrylic resins, which have promising potential in the application of nail polish.

## 1. Introduction

With the improvement of human living standards, more and more people pay attention to nail care and modification. In this aspect, UV-curable nail polish has been loved by consumers for its rapid film formation and durable characteristics. It was noting that UV-curable resin is an important and basis material to obtain UV-curable nail polish. However, UV-curable resins commonly originate from fossil resources, and their related properties create challenges for their widespread application, such as their high viscosity, insufficient mechanical properties and thermostability. Although active diluents are often introduced to reduce the viscosity of photocured resins, it is difficult to improve mechanical toughness and thermal stability and maintain the safety of resin-based films at the same time [1]. For instance, many active diluents such as toluene and styrene are harmful to the human body [2]. Therefore, it is of great significance to design and synthesize a bio-based resin with good thermal stability, low viscosity, low irritation and good mechanical properties for nail polish application.

Soybean oil-based acrylate, as one kind of soybean oil derivative, is synthesized by the acrylation of epoxidized soybean oil [3], which has high activity with the groups containing C=C groups, hydroxyl groups and an ester group. And because of the advantages of low viscosity, low irritation and high photosensitivity, soybean oil-based acrylate is a kind of ideal UV-curable resin matrix, which has already been industrialized and can be purchased and used in the market. Therefore, soybean oil-based acrylate is a promising candidate to replace fossil-based UV-curable resin. For instance, the Mendes-Felipe group reported the high UV-curing activity of acrylate epoxidized soybean oil and could prepare an acrylate-cured product with the designed shape via digital light processing [4]. However, the related cured product displayed ordinary elongation at a break of less than 50%. Thus, it is very necessary to develop new soybean oil-based acrylates and their derivatives for the preparation of UV-curable resins with high strain.

In this study, a soybean oil-based acrylate (AESO) photosensitive resin was synthesized by reacting epoxidized soybean oil with acrylic acid and then blended with anhydride derivatives. The Fourier transform infrared spectroscopy (FT-IR) and **proton nuclear magnetic resonance** (^1^H NMR) structures of the AESO resin were characterized. And the effects of the anhydride derivative addition on the viscosity property of AESO and the thermal stability and tensile properties of the AESO-based cured film were investigated. Moreover, isophorone diisocyanate (IPDI) was introduced into AESO to obtain soybean oil-based polyurethane acrylate, which can further improve the resin’s mechanic properties. Thereafter, soybean oil-based polyurethane acrylate was blended and compounded with the anhydride derivatives, and a series of photosensitive polyurethane resin-based cured films were prepared, which had a favorable application prospect in the nail polish products. The effects of the addition of IPDI, viscosity, heat resistance, alkali solubility, and flexibility on the cured film properties were investigated.

## 2. Experiment Section

### 2.1. Materials

Maleic anhydride (MA), epoxy soybean oil (ESO), and acrylic acid (AA) were sourced from Shanghai McLean Biochemical Technology Co., Ltd., in Shanghai, China. Isophorone diisocyanate (IPDI) and hydroxyethyl acrylate (HEA) were procured from Guangzhou Boxing Chemical Technology Co., Ltd., in Guangzhou, China. Methyl tetrahydro phthalic anhydride (MTHPA) was provided by Guangzhou Zhonggao Chemical Co., Ltd., in Zhonggao, China. 2-hydroxy-2-methylpropiophenone (PI-1173) was bought from BASF Co., Ltd., in Shanghai, China. All the chemicals were utilized in their as-received state without undergoing any purification process.

### 2.2. Synthesis of Anhydride Derivatives

**Scheme 1:**
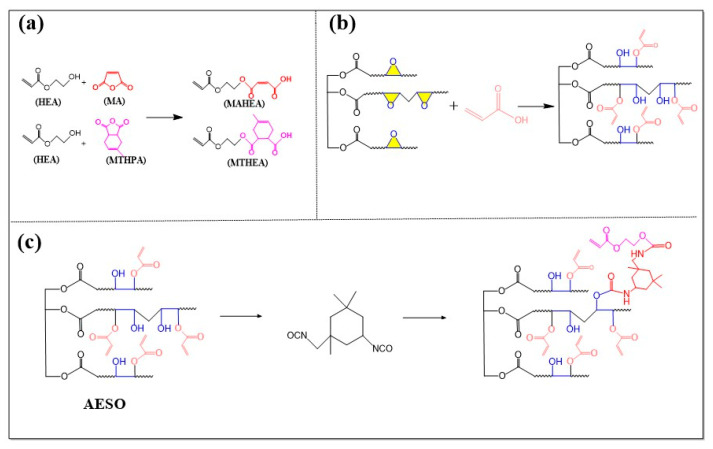
Synthesis routes of (**a**) anhydride derivatives; (**b**) epoxidized soybean oil-based acrylic photosensitive resin (MATEO) and (**c**) photosensitive polyurethane resin (IPM).

The synthetic routes for the derivatives of MA and MTHPA are displayed in Scheme 1a. For the synthesis of the MA derivative (named MAHEA), according to the previous studies [5], 42.23 g of MA was placed into a 250 mL triple-necked flask and then mixed with 50 g of HEA. After being heated to 90 °C, the mixture underwent an esterified reaction between with anhydride and HEA (an MA and HEA molar ratio of 1:1) under the stirring speed of 250 rpm until the FT-IR characteristic peak of anhydride disappeared. Moreover, the MTHPA derivative (named MTHEA) was synthesized by the same preparation procedure but using MTHPA (71.55 g) instead of MA as the anhydride material.

### 2.3. Preparation of Soybean Oil-Based Acrylates (AESO) and Polyurethane Acrylate (IPM)

With reference to previous studies [6], the detailed preparation procedure of AESO is displayed as follows. At first, 48.0 g of ESO was mixed into a 250 mL triple-necked flask containing 14.2 g of AA. Afterwards, the mixture system underwent a ring-opening esterification reaction with a stirring speed of 250 rpm at 110 °C until the acid value of the reaction system no longer changed. Herein, AA was in excess, and after the esterification reaction of ESO and AA, the acid value dropped to 28–29 mg KOH∙g^−1^, which was the end point of the reaction. Moreover, the AESO liquid product with a light-yellow color was gained. The formation route of AESO is demonstrated in Scheme 1b.

The synthesis route of the soybean oil-based polyurethane acrylate (IPAE resin) is displayed in Scheme 1c. Specifically, AESO (50 g) and IPDI (2 wt%, 3 wt%, and 5 wt%, with respect to the total content of AESO) were added and mixed in a 250 mL triple-necked flask. Then, the mixture was heated to 50 °C and was continuously stirred at 250 rpm until the disappearance of the FT-IR characteristic peak of NCO, which originated from IPDI. It is worth noting that IPAE resins prepared with 2 wt%, 3 wt%, and 5 wt% of IPDI were regarded as IPAE-2 resin, IPAE-3 resin, IPAE-5 resin, respectively.

### 2.4. Construction of Soybean Oil-Based UV-Cured Coating Films

For the preparation of the soybean oil-based acrylate cured coating film, a certain amount of AESO resin, MAHEA and MTHEA anhydride derivatives were mixed and produced the soybean oil-based acrylate photosensitive resin (named MATEO resin). And the specific contents of AESO, MAHEA and MTHEA are shown in Table 1. Moreover, with reference to previous studies [7], MATEO was mixed with photoinitiator 1173 (5 wt% of the total mass of the mixture system) and then stirred with a speed of 250 rpm for 30 min at room temperature and afterwards defoamed by ultrasonication at 150 W for 20 min to obtain the coating precursor. Furthermore, the coating precursor was uniformly coated on a glass plate using a 500 μm four-side preparator and then irradiated by a 5 kW UV mercury lamp for 40 s to form the soybean oil-based acrylate-cured coating film (named the MATEO film).

For the fabrication of soybean oil-based polyurethane acrylate-cured coating film, the IPAE resin, MAHEA and MTHEA anhydride derivatives were mixed and we obtained the soybean oil-based polyurethane acrylate photosensitive resin (regarded as IPM resin). Herein, the specific use contents of IPM, MAHEA and MTHEA were 50 wt%, 16 wt% and 34 wt%, respectively. And the IPM film was produced by adding different proportions of isocyanate in the AESO resin. In addition, the IPM films prepared with IPAE-2 resin, IPAE-3 resin, and IPAE-5 resin were named IPM-2 film, IPM-3 film, and IPM-5 film, respectively. Furthermore, the control AESO coating film was also prepared by the same preparation procedure but only using the AESO resin and without MAHEA and MTHEA anhydride derivatives.

### 2.5. Characterization

FT-IR tests of the samples were carried out on a Thermo-Nicolet iS10 FT-IR spectrometer in the scanning range from 4000 to 400 cm^−1^ with a resolution of 2 cm^−1^ for 32 scans.

^1^H nuclear magnetic resonance analysis (^1^H NMR) spectra of the samples were determined by a Bruker AV600 spectrometer using CDCl_3_ as the solvent, and we scanned the samples eight times at a magnetic field strength of 400 MHz with the nuclear magnetic resonance instrument.

The acid values of AESO, MATEO and IPAE were subjected to testing in accordance with the specific procedures detailed in GB/T 2895–1982. Firstly, precisely 1.000 g of the sample was taken and combined with 15 mL of ethanol solution within a conical flask. The contents of the flask were then vigorously shaken until the sample was entirely dissolved, thereby generating the sample solution. Subsequently, a quantity of 4 to 5 drops of phenolphthalein indicator was carefully introduced into the very same conical flask. Subsequently, the test solution was titrated with 0.5 mol·L^−1^ KOH/ethanolic solution (28 g of KOH solid powder dissolved in 1000 mL anhydrous ethanol solution). The acid value (Ac) was computed in accordance with Formula (1) as follows:


(1) $$\mathrm{Ac}=\frac{56.1\times C\times V}{M}$$

In this context, 56.1 stands for the molecular weight of KOH. *C* denotes the normality concentration of the KOH solution, which is measured in mol·L^−1^. *V* represents the volume of the KOH solution consumed during the titration, with the unit of mL. *M* refers to the weight of the sample being tested.

The isocyanate value test of IPAE was executed according to the procedures specified in HG/T 2409-1992. To be specific, 0.2 mol·L^−1^ di-n-butylamine-toluene solution was prepared as follows. In total, 25.85 g of di-n-butylamine was dissolved in toluene, transferred into a 1000 mL volumetric flask, diluted with toluene, and then shaken well. Then, about 2 g of the sample was weighed and placed in a conical flask, mixed with 20 mL of toluene and 10 mL of toluene-dibutylamine solution to dissolve the IPM sample completely. Afterwards, 50 mL of isopropanol and 3–4 drops of bromocresol green indicator were added into the above conical flask, and then the mixture was titrated with 0.1 mol∙L^−1^ hydrochloric acid standard solution. After seeing the yellow color of the sample solution and keeping the color without change for 15 s, the reaction was regarded as having arrived at the endpoint. In addition, the blank group (without a sample, and other steps are the same) was also tested by the same preparation procedure of the sample group at the same time. The isocyanate value was calculated based on Formula (2) as follows:

(2) $$W_{\left(-NCO\right)}=\frac{(V_{0}-V_{1})\times C\times 42}{1000m}$$

where *V*_0_ means the volume of hydrochloric acid standard solution consumed in the blank experiment (mL); *V*_1_ is the volume of hydrochloric acid standard solution consumed in the experimental samples; *C* presents the molar concentration of hydrochloric acid standard solution (mol·L^−1^); 42 is the molar mass of isocyanate groups (g·mol^−1^); and *m* displays the mass of the sample (g).

The rheological viscosity characteristics of the MATEO, AESO, IPAE and IMP resins were examined using an Anton Paar MCR 502 rheometer. The test was carried out at 30 °C with the shear rate varying from 0.01 s^−1^ to 100 s^−1^. The rotor with a diameter of 25 mm and an angle of 2° was selected. The separation distance between the two flat plates was set at 0.104 mm.

The mechanical performances of the UV-cured coating films were carried out by a UTM 4204 electronic universal machine. To be specific, the sample dimensions were 40.0 mm (length) × 10.0 mm (width) × 0.3 mm (thickness), and the tensile tests was performed at a tensile rate of 10 mm·min^−1^. Each sample was fixed on a mold and the tensile load was applied along the longitudinal axis until the rectangular specimens ruptured. Five measurements were made for each group to ensure the accuracy of the data, and the average value was calculated to obtain the tensile strength and elongation at break data of the cured film.

The solvent resistance property of the cured coating films was subjected to testing procedures in three distinct solvents, namely water, ethanol and toluene. To commence this process, the film samples were carefully immersed into sealed glass bottles which were pre-filled with the aforementioned solvents. These samples were then left to soak in these solvents at normal room-temperature conditions for a period of 48 h. After the soaking period was completed, the wet film samples were taken out and placed inside an oven, which was set at a temperature of 60 °C. The samples were kept in the oven until they were completely dried and reached a state where their weight became constant. The weight ratio (*WR*) of the residual cured film was obtained in line with the following Formula (3):

(3) $$WR=\frac{M_{a}}{M_{0}}\times 100\%$$

where *M*_a_ represents the weight of the cured film after soaking and drying, while *M*_0_ is the original weight of the cured film before soaking.

The thermogravimetric analysis (TGA) was implemented with the utilization of the Netzsch TG209 F1LibraTM thermogravimetric analyzer. The temperature regime was defined to span from 35 °C to 700 °C, during which the heating was effected at a rate of 10 °C∙min^−1^, and the entire analytical process was carried out within an environment consisting of nitrogen gas.

A dynamic mechanical analysis (DMA) of the UV-cured films was carried out using the Netzsch DMA 242E dynamic mechanical analyzer in the tension mode and at a frequency of 1 Hz. In the DMA tests, the samples, which measured 25.0 mm in length, 6.0 mm in width and 0.3 mm in height, were cooled down to −60 °C with liquid nitrogen and then heated up to 160 °C at a heating rate of 5 °C min^−1^.

The adhesion test of the cured films was conducted by a fully automated pull-apart adhesion tester with a range of 0.7–20 MPa. The samples were uniformly coated on A3 iron sheets and cured by pulling the film using a 500 μm four-side preparator, and then the spindles were glued to the cured film and left to stand for 24 h before being tested.

The gloss test of the cured films was performed by using a gloss tester (MG60S) to investigate the gloss value of the cured film.

The gel content test of the cured films was carried out to examine the degree of the curing of the film. To be more precise, the cured film was immersed in a sealed glass bottle filled with 20 mL of acetone and left to soak for 48 h at room temperature. Subsequently, the film was dried in an oven set at 60 °C until it reached a constant weight. The gel content (*GC*) of the cured film was calculated by the following Formula (4):

(4) $$GC=\frac{W_{a}}{W_{0}}\times 100\%$$

where *W*_a_ is the mass of the film samples after drying in the oven, and *W*_0_ is the original mass before soaking in the solvents.

The acid and alkali resistance assays of the cured films were implemented within sealed glass containers filled with 10 wt% sodium hydroxide solution and 10 wt% hydrochloric acid solution for a duration of 48 h. The morphological characteristics of the cured film both before the soaking process and after the drying treatment were observed and analyzed, respectively.

## 3. Results and Discussion

### 3.1. Synthesis and Characterization of Anhydride Derivatives and Resins

**Figure 1:**
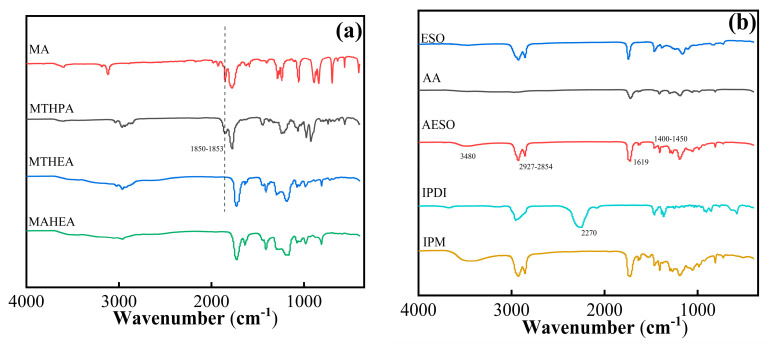
FT-IR spectra of (**a**) MA, MTHPA, MTHEA, MAHEA, MATEO and (**b**) ESO, AA, AESO, IPDI, MATEO, IPM.

As shown in Figure 1a, in the FT-IR spectra of MA and MTHPA, the peaks at 1850 cm^−1^ and 1853 cm^−1^ belong to the stretching vibration of the anhydride. The disappearance of the anhydride peaks in the FT-IR spectra of MTHEA and MAHEA after esterification indicate that MA and MTHPA successfully react with HEA, respectively. In the FT-IR spectrum of AESO in Figure 1b, the peak at 1619 cm^−1^ represents the C=C stretching vibration derived from the acrylate groups [5], the peak at 3480 cm^−1^ is ascribed to the hydroxyl group peaks, and the peaks at 2927–2854 cm^−1^ are methyl and methylene characteristic manifestations, which indicate that the ring-opening esterification reaction of the epoxy groups from ESO and AA occurred to form hydroxyl groups [8]. At the same time, in the FT-IR spectrum of IPDI, the characteristic peak at 2270 cm^−1^ is attributed to the stretching vibration of the -NCO groups. And in the FT-IR spectrum of IPAE appears a new peak at 1530 cm^−1^, which originates from the vibrational peak of an amide in the carbamate groups, and it displays the -NCO peak at 2270 cm^−1^ (ref. [9]), which indicates that the polyurethane successfully synthesizes on the AESO structure to obtain IPAE resin.

**Figure 2:**
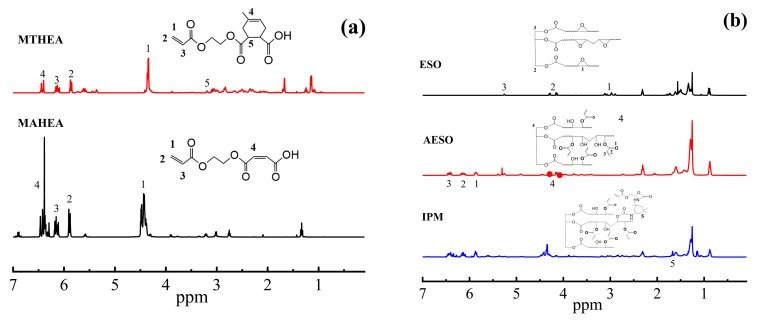
^1^H NMR spectra of (**a**) MTHEA, MAHEA and (**b**) ESO, AA, AESO, IPM.

The chemical structures of anhydride derivatives and resins were further investigated by the ^1^H NMR spectrum analysis. As shown in Figure 2a, the peak around 5.85–6.25 ppm is ascribed to the double-bonded proton peak of HEA [10], the peak at 6.40–6.42 ppm is attributed to the double-bonded peak of MA, and the peak at 3.20 ppm is the tertiary-hydrogen characteristic chemical shift of MTHPA. The above results indicated that HEA successfully reacted with MA and MTHPA to prepare anhydride derivatives, respectively. Moreover, it is observed from Figure 2b that in the ^1^H NMR spectrum of AESO, the -CH characteristic peaks of the epoxy group almost disappears at 2.8–3.1 ppm, and three sets of characteristic peaks at 5.8–6.5 ppm appear, which are assigned to the AA-based protons [11] and in turn indicate the successful reaction of the epoxy group of ESO and AA. In addition, the ^1^H NMR spectrum of IPAE show isocyanate methylidene proton peaks at 1.57–1.59 ppm, which indicate that the IPDI is successfully introduced into the AESO structure to obtain the polyurethane acrylate resin IPAE.

### 3.2. Viscosity Analysis of MATEO and AESO Resins

**Figure 3:**
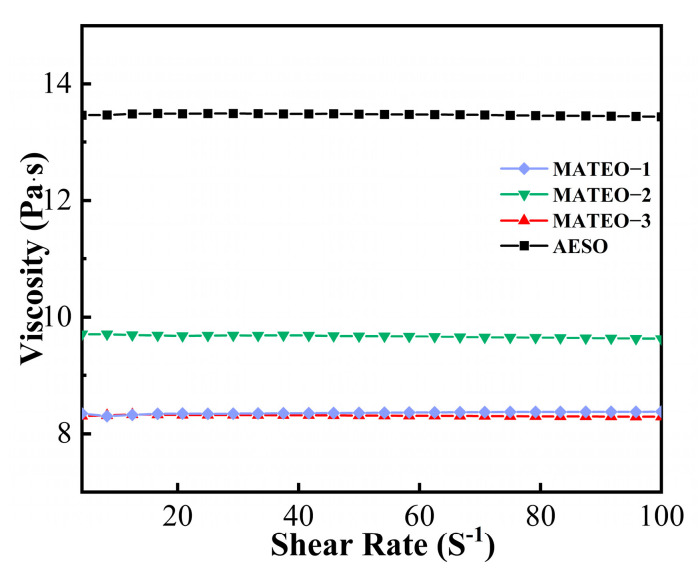
Rheological viscosity analysis of MATEO and AESO.

As observed from Figure 3 and Table 2, the viscosity of AESO is 13.48 Pa∙s, which is the highest viscosity in the four tested resins. After adding the anhydride derivatives into AESO, the MATEO resins display reduced viscosity, which is ascribed to the low viscosity of anhydride derivatives. Specifically, among the three different ratios of MATEO resins, the MATEO-3 resin had the lowest viscosity of 8.31 Pa∙s.

The low viscosity may be due to the small molecular structure of the anhydride derivatives, which have polar carbonyl or other groups that are able to be inserted into the macromolecular system either chemically or physically, thus reducing the intermolecular interaction forces. As the interactions of the molecular chains are weakened, the overall fluidity of the system increases, leading to a decrease in viscosity. Moreover, the hydrolyzed products of anhydride derivatives, which contain both hydrophobic styrene and hydrophilic anhydride chains, are often used as polymer dispersants [12]. The low viscosity of the resin assisted in endowing the good fluidity of the resin. Therefore, the addition of anhydride derivatives could contribute to the reduction in the viscosity of the resin system. Low-viscosity resins had favorable workability and application areas, which availed the application performance of the MATEO resin to broaden the application of bio-based materials.

### 3.3. Mechanical Properties of MATEO and AESO Cured Films

**Figure 4:**
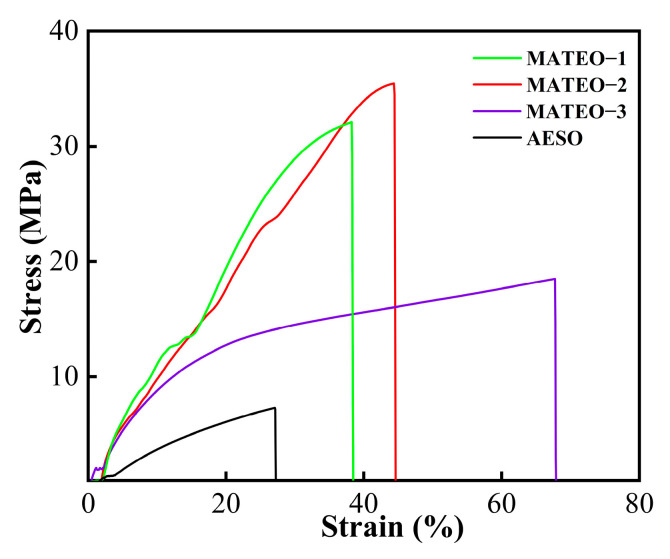
Stress–strain curves for different ratios of MATEO.

As observed in Figure 4 and Table 2, the elongation at break and tensile strength of the MATEO cured films increased for all ratios of cured films compared to the AESO cured films. The MATEO-2 cured film had the best overall mechanical properties, with an elongation at break of 44.32% and a tensile strength of 35.36 MPa, and the related elongation at break and tensile strength were 1.64 and 4.90 times greater than those of the AESO cured film, respectively. The overall elongation at break of these three ratios of MATEO resins ranged from 31% to 68%. In a previous study, anhydride derivatives were found to have lower Young’s modulus and ultimate tensile strengths with higher ultimate strains compared to DGEBA-based epoxy monomers. They also exhibited highly enhanced properties compared to conventional DGEBA-based epoxy resin systems, with more than three times higher stresses and strains [13]. In conclusion, the addition of appropriate amounts of anhydride derivatives into AESO resin could improve the mechanical properties of the AESO-based cured films. In practical application, the ratio of AESO and anhydride derivatives could be adjusted according to the actual application needs in order to achieve the application performance of AESO-based UV-curing materials.

### 3.4. Thermogravimetric Assessment of MATEO and AESO Cured Films

**Figure 5:**
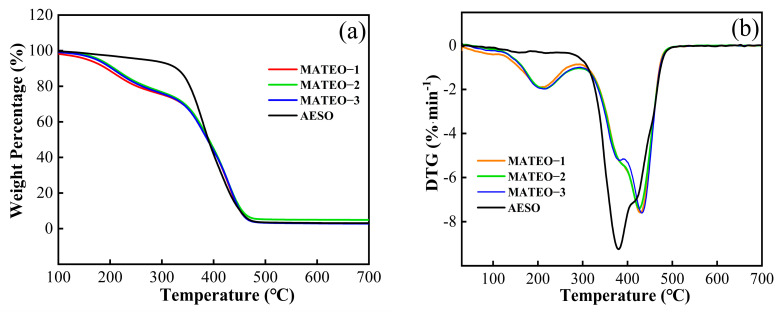
(**a**) TGA and (**b**) DTG curves of MATEO and AESO cured coating films.

The thermal stability of the cured films was analyzed by thermogravimetric testing. The TGA and DTG curves are shown in Figure 5. The thermal decomposition temperatures and residual charcoal rates of the cured films with mass losses of T_10%_ and T_50%_ are shown in Table 3. Except for the AESO cured film, the T_10%_ data of all MATEO cured films were around 200 °C. Moreover, there was no significant difference between the T_10%_ and T_50%_ data of all cured films. As observed from Figure 5, it can be observed that the curves of the AESO cured film and the MATEO cured films display different thermal degradation behaviors. In the AESO cured film system, the first decomposition stage had a slower decrease, which was mainly caused by the volatilization of small molecules such as catalysts and the remaining photoinitiators. And as the temperature exceeded 300 °C, the decomposition of soybean oil fatty chains caused a sharp decrease in the weight of the AESO cured film, and then, until the temperature approached around 500 °C, the weight of the AESO cured film showed almost no clear change [14]. Moreover, the TGA and DTG curves of all MATEO cured films in the thermal degradation process displayed two distinct pyrolysis stages. In the first pyrolysis stage between around 130 and 300 °C, an obvious weight loss was generated by the decomposition of raw materials including photoinitiators and catalysts. The second decomposition stage mainly occurred between 300 and 470 °C and tended to stabilize at 500 °C, which might have been due to the decomposition of the long fatty chain of the crosslinked mixture and the carbonization effect. In addition, the main degradation temperature of the AESO cured film was 380 °C, while the maximum degradation temperature of the MATEO cured films was 430 °C. This might be due to the fact that the ester group and other characteristic structures in the anhydride derivative molecules could have improved the thermal stability of the MATEO cured films [15].

### 3.5. Dynamic Thermomechanical Study of MATEO and AESO Cured Films

**Figure 6:**
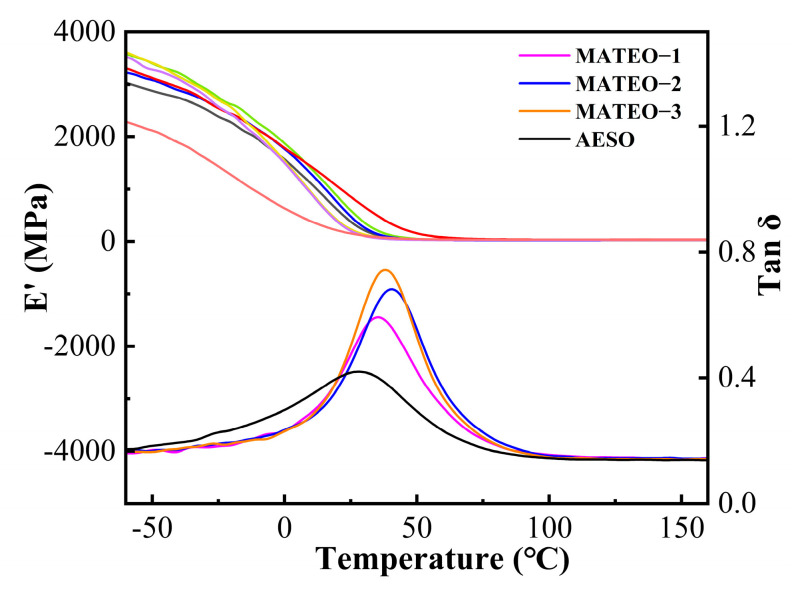
Energy storage modulus and tan δ curves for MATEO and AESO cured films.

The dynamic thermomechanical properties of the MATEO and AESO cured films were investigated by a DMA analyzer. The energy storage modulus (*E*′) and loss coefficient (tan δ) curves of the MATEO and AESO cured films are plotted in Figure 6. The maximum peak in the tan δ curve is related with the glass transition temperature (T_g_) of the cured films. As seen from Figure 6, there is only one tan δ peak and T_g_ peak in all the cured films, which indicates the good compatibility of the cured films. While the stiffness of the cured films can be evaluated by E′_25_, the thermodynamic behavior is closely related to the crosslinking degree. According to previous research, the crosslinking degree has an influence on the crosslinking characteristics of the cured film: as the temperature rises, the mechanical strength of the cured film gradually increases [16]. According to the theory of rubber elasticity, the crosslink density (*V*_e_) of the cured film can be calculated using the following formula:

$$V_{e}=\frac{E\prime }{3RT}$$

*E*′ corresponds to the energy storage modulus of the cured film, *T* is the absolute temperature of the cured film in the rubberized state (T_g_ + 30 °C), and *R* is the gas constant. As observed from Table 3, the T_g_ value of the cured film increases from 28 °C to 40.5 °C with the addition of anhydride derivatives; meanwhile, the *E*′_25_ value increases from 151.14 MPa to 593.82 MPa, which shows that the addition of anhydride derivatives could increase the T_g_ and rigidity of the AESO-based cured film system [17]. To summarize, the addition of an appropriate amount of anhydride derivatives could significantly improve the rigidity and T_g_ value of the cured film. Therefore, one could choose different ratios of anhydride derivatives and AESO according to the different application needs of the film products.

### 3.6. Adhesion and Gloss Evaluation of MATEO and AESO Cured Films

The adhesion and gloss results of the MATEO and AESO cured films are shown in Table 4. As observed, after the addition of anhydride derivatives, the adhesion and gloss results markedly increased; the adhesion especially reached the highest values of 1.03 MPa for the MATEO-2 cured film, which was as much as 3.68 times as that of the AESO cured film. Moreover, the gloss level of all cured films was above 100. The MATEO-2 cured film displayed the highest value of 133.1, which indicated that the surface of the MATEO-2 cured film was smooth, with good gloss performance. Moreover, the adhesion is related to the -OH content of the coating. The enhanced adhesion may be related to the hydrogen bonding generated in the carboxyl groups in the anhydride derivatives and in the hydroxyl groups in AESO [18].

### 3.7. Gelation Rate, Solvent Resistance and Acid/Alkali Resistance of MATEO and AESO Cured Films

The gelation rate, solvent resistance, and acid/alkali resistance results of the cured films are shown in Table 4. The gelation rate of all the cured films was above 88%. All cured films showed excellent solvent resistance, which was due to the structure of the hydrophobic fatty chain of soybean oil [19]. The fatty chains in soybean oil are long-chain hydrocarbon groups that are significantly hydrophobic. These hydrophobic chain segments can form dense non-polar regions in the material, thereby reducing the penetration of polar or weakly polar solvent molecules. The uniform distribution of the fatty chains in the crosslinked network effectively shields the crosslinking points, further improving solvent resistance. Moreover, it was seen that all the cured films displayed high weight retention soaking in the solvent, displaying favorable solvent resistance. In addition, the AESO cured films were not alkali-soluble [20], but after the addition of the anhydride derivatives, all the MATEO cured films showed a whitening and swelling of the film surface, which might have been due to the many ester bonds that could react with the alkali, and this would be beneficial for the recovery and removal of the cured films after usage.

### 3.8. Acid Value and Rheological Viscosity Testing of IPM Resins

**Figure 7:**
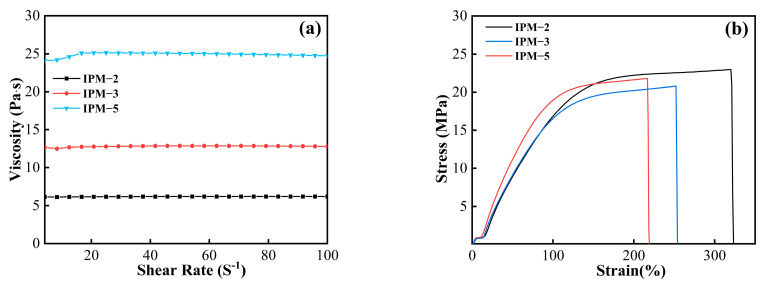
Rheological viscosity curves of (**a**) IPM resins; (**b**) stress–strain curves of IPM cured coating films.

The rheological viscosities of the IPM resins are shown in Figure 7a, and the acid value and viscosity results are shown in Table 5. As seen from Figure 7a, it can be observed that the viscosity of the IPM-2 resin is the lowest, which is 6.2 Pa∙s. With the increased content of isocyanate IPDI, the viscosity of the IPM resin also increased, and the highest viscosity reached 25.0 Pa∙s. This result indicated that the addition of isocyanate was an obviously important factor for the increased viscosity of the IPAE-based resin. When IPDI reacts with AESO, it increases the viscosity of the resin. As the amount of IPDI increases, the viscosity decreases. The viscosity of a resin system depends mainly on the resin components and their intermolecular interactions, which are related to the resin components and their intermolecular interactions [21]. In the production and synthesis of materials, the viscosity of the IPAE-based IPM resin system could be changed by adjusting the isocyanate content. Table 5 shows that the acid value results of the IPM resin are generally consistent with those for the MATEO resin, with no big difference between the two acid values of the IPM and MATEO resins, which in turn indicates that the -NCO group of diisocyanate mainly reacts with the -OH from the AESO, and the addition of a certain amount of isocyanate does not influence the acid value of the related resin system.

### 3.9. Mechanical Property Investigation of IPM Cured Films

The mechanical properties of the IPM cured films are shown in Figure 7b. The tensile strengths of all three cured films were above 20 MPa, and the elongation at break was up to 320%. It is worth noting that the tensile strengths of the IPM-2, IPM-3 and IPM-5 films were 22.89 MPa, 20.81 MPa and 21.77 MPa, respectively, and the related elongations at break were 320%, 252% and 216%, respectively. Compared with the MATEO cured film, the elongation at break was significantly improved. In particular, the elongation at the break of the IPM-2 cured film was 4.72 times higher than that of the MATEO-3 cured film (67.80%). This result indicated that the addition of the isocyanate visibly increased the elongation at the break of the cured film and in turn assisted in improving the toughness of the cured film. This result might be because that the NCO group was capable of generating polyurethanes containing dynamic urea bonds with AESO, and the tunability of its chemical composition and microphase-separated structure could provide the material with ultra-high ultimate strength and superb toughness [22]. Therefore, the introduction of the polyurethane structure in the IPM resin increased the toughness of the cured film, which endowed the related cured film application potential in the field of high toughness requirements.

### 3.10. Thermogravimetric and Dynamic Thermomechanical Analysis of IPM Cured Films

**Figure 8:**
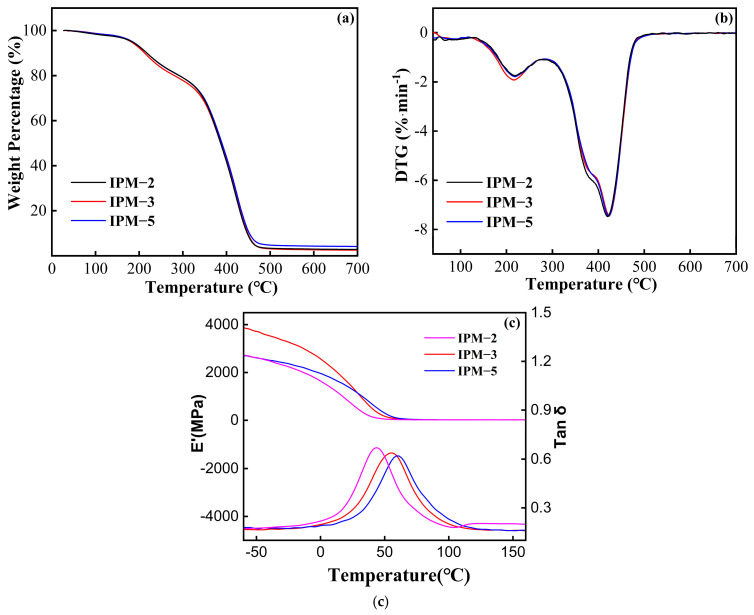
(**a**) TGA, (**b**) DTG and (**c**) energy storage modules, loss factor curves of IPM cured coating film.

The TGA, DTG, energy storage modulus and loss factor curves of the cured films are shown in Figure 8a,b. It could be observed that the TGA and DTG curves of all IPM cured films showed the same trend, which mainly included two thermal degradation stages. The first stage mainly occurred between 160 and 300 °C, which was mainly due to the mass loss caused by photoinitiator 1173, the catalyst and trace isocyanate residues. The second stage occurred between 300 °C and 480 °C, which was mainly due to the long carbon chain structure and carbonization. And the specific data of the T_10%_, T_50%_ and residual carbon rate are shown in Table 6. There is no significant difference in the mass loss of the cured films except for the IPM-5 film, which has a residual carbon rate of 4.14%. The T_10%_ values of the three IPM cured films are around 217 °C, and the T_50%_ values are between 386 and 389 °C, indicating that the addition of isocyanate does not affect the thermal stability of the IPM cured films. The maximum decomposition temperature of the IPM cured films appeared around 420 °C, which showed the favorable thermal stability of the IPM cured films.

The energy storage modulus and loss factor curves of the cured film are shown in Figure 8c. It is shown that the cured film has only one tan δ peak and Tg peak, indicating that all the resins in the cured films are homogeneous and compatible. Table 6 shows that the IPM-5 cured film has the largest T_g_ value, reaching 60 °C with increasing isocyanate content. As the isocyanate content increased, the T_g_ and E′25 values of the cured film show an increasing trend, which indicates that the addition of an appropriate amount of isocyanate could improve the T_g_ value and rigidity of the cured film. According to the theory of rubber elasticity, the crosslink density (Ve) of the system can be calculated. From Table 6, it can be seen that there is no significant difference in the crosslink density of all cured films, probably because the addition content of IPDI is between 2% and 5%, which does not clearly affect the performance effect of the cured film crosslinking degree. In response to the above conclusion, different contents of isocyanate could be selected for chemical modification according to the actual needs.

### 3.11. Comprehensive Performance Analysis of IPM-Based Cured Films

The comprehensive performance of the IPM-based cured films is shown in Table 6. All three cured films achieved a gelation rate of more than 90% and solvent resistance of more than 94%, and the cured films also showed excellent acid resistance. In addition, all the cured films showed alkali solubility due to the addition of anhydride derivatives, which resulted in a large number of carboxyl and ester groups in the system that could react with alkalis. In conclusion, the IPM-5 cured films displayed a balanced overall performance.

**Figure 9:**
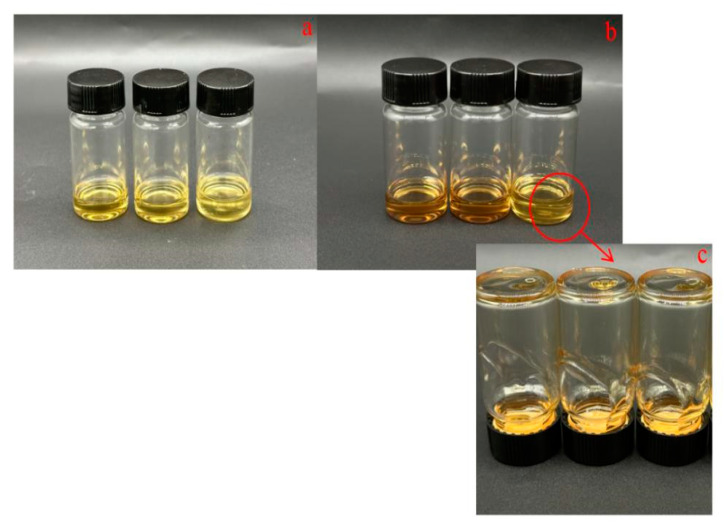
(**a**) Original state of static samples; (**b**) front view of the samples’ appearance after 7 d in the oven at 90 °C; (**c**) inverted view of the appearance after 7 d in the oven at 90 °C.

From Figure 9, the state of appearance and morphology of the liquid resin can be observed. The IPM-2, IPM-3, and IPM-5 resins are dispensed in glass bottles from left to right, and at this time, all IPM resins are in a liquid flow state with a light-yellow color. It can be observed that the color of all the resins shows different degrees of deepening after 7 d in the 90 °C oven, and the color of IPM-5 is slightly lighter than that of the other two resins. And the resins are still fluid after 7 d of baking at high temperatures. These results show that all IPM resins have superior thermal stability and heat resistance. In addition, the IPM-5 resins have excellent yellowing resistance, which is important for improving safety and broadening the range of epoxy resin applications. In addition, the IPM-5 resin also has excellent yellowing resistance, which is of great significance to improve the safety of production and broaden the application scope of biomass-based resin in various fields.

## 4. Conclusions

Hydroxyethyl acrylate was esterified with maleic anhydride and methyl tetrahydrophthalic anhydride to produce anhydride derivatives, and then the anhydride derivatives were ring-opening reacted. Moreover, a soybean oil-based acrylic photosensitive resin was synthesized. It was shown that the related UV-curable resin had excellent thermal stability and a reduced viscosity of 8.31 Pa∙s. Meanwhile, the related UV-cured films were prepared, which had the tensile strength of 35.36 MPa and elongation at the break of 67.8%. In addition, the isophorone diisocyanate was introduced into soybean oil-based acrylate to obtain polyurethane acrylate, which could be thermally stable at 90 °C for 7 d, and the elongation at break of the related cured films was significantly improved up to 320%. This work provided a solvent-free approach to prepare bio-based polyurethane acrylate resins, which were suitable to apply in nail polish products. And the established approach of bio-based UV-curable resins with universal and various applicability can be readily broadened to apply to the large-scale production of resin construction. However, the removal of the soybean oil-based polyurethane acrylate cured film generally needed to use an alkali solution. Thus, further exploration and thought is needed in terms of removal methods regarding nail polish removal or resin flaking.

## Figures and Tables

**Table 1:** Synthesis ratio and acid value of MATEO and AESO resins.

Resin	AESO (wt%)	MAHEA (wt%)	MTHEA (wt%)	Ac (mg KOH·g^−1^)
MATEO-1	50	25	25	118.62
MATEO-2	50	16	34	123.49
MATEO-3	50	12	38	121.24

**Table 2:** Viscosity and cured film mechanical properties of MATEO and AESO resins.

Resin	Viscosity (Pa·S)	Stress (MPa)	Strain (%)
MATEO-1	8.36	32.09	31.98
MATEO-2	9.67	35.36	44.32
MATEO-3	8.31	18.51	67.80
AESO	13.48	7.21	27.05

**Table 3:** Gel content and solvent resistance properties of the cured films.

Film	T_g_ (°C)	E′_25_ (MPa)	E′_Tg+30 °C_ (MPa)	V_e_ (mol·m^−3^)	T_10%_ (°C)	T_50%_ (°C)	W_char_ (%)
MATEO-1	35.3	381.41	26.85	3180.67	192.62	389.42	3.02
MATEO-2	40.5	593.82	27.24	3178.04	208.21	208.21	4.80
MATEO-3	38.1	479.43	22.61	2254.02	203.24	388.04	2.77
AESO	28.0	151.14	39.75	4812.62	328.37	389.17	3.09

**Table 4:** Comprehensive performance of MATEO, AESO and IPM cured films.

Film	GC	Glossiness	Adhesion	Acid Resistance	Alkali Resistance	Solvent Resistance (%)		
	(%)	(%)	(MPa)			Toluene	Ethanol	Water
MATEO-1	91.04	126.9	0.40	Unchanged	Dissolved	95.44	97.79	99.62
MATEO-2	97.44	133.1	1.03	Unchanged	Dissolved	90.34	93.53	94.67
MATEO-3	88.75	118.5	0.70	Unchanged	Dissolved	99.61	93.07	95.45
AESO	95.79	122.3	0.28	Unchanged	Unchanged	99.57	95.95	99.01
IPM-2	91.10	68	0.6	Unchanged	Dissolved	98.64	95.36	94.94
IPM-3	93.58	114.90	0.86	Unchanged	Dissolved	99.82	97.51	97.83
IPM-5	97.64	112.60	0.81	Unchanged	Dissolved	97.97	99.84	99.14

**Table 5:** Synthesis ratios and acid values of IPM resins.

Resin	AESO-IPDI	IPDI Usage (%)	MAHEA (wt%)	MTHEA (wt%)	Acid Value (mg KOH·g^−1^)
IPM-2	50	2%	16	34	115.50
IPM-3	50	3%	16	34	111.70
IPM-5	50	5%	16	34	112.50

**Table 6:** Comprehensive property details of IPM-based cured coating film.

Film	T_g_ (°C)	E′_25_ (MPa)	E′_Tg+30 °C_ (MPa)	V_e_ (mol·m^−3^)	T_10%_ (°C)	T_50%_ (°C)	W_char_ (%)	Stress (MPa)	Strain (%)
IPM-2	43.70	658.99	23.04	2663.24	217.42	386.42	2.44	22.89	320
IPM-3	55.40	1378.28	24.75	2767.54	212.67	386.17	2.52	20.81	252
IPM-5	60.00	1284.84	24.56	2606.99	217.57	389.57	4.14	21.77	216

## Data Availability

The data presented in this study are available on request from the corresponding author.
